# Alveolar Bone Quality in Individuals With Cleft Lip and Palate With Missing Lateral Incisors: Orthodontic Space Closure Versus Space Opening

**DOI:** 10.1177/10556656241312499

**Published:** 2025-01-23

**Authors:** Malak Aldosari, Jay Shah, Jaemin Ko, Snehlata Oberoi

**Affiliations:** 1 Department of Orthodontics and Dentofacial Orthopedic Dental Hospital, 149994 King Saud Medical City, Riyadh, Saudi Arabia; 2 School of Dentistry, 8785 University of California, San Francisco, CA, USA; 3 Craniofacial and Special Care Orthodontics, Division of Dentistry, Children's Hospital Los Angeles, Los Angeles, CA, USA; 4 Department of Orofacial Sciences and Orthodontics, Division of Craniofacial Anomalies, School of Dentistry, University of California, San Francisco, CA, USA

**Keywords:** cleft lip and palate, orthodontics, tooth movement, tooth agenesis, bone grafting

## Abstract

**Objective:**

The purpose of this study was to quantitatively assess the alveolar bone support of teeth adjacent to the cleft site in individuals with nonsyndromic cleft lip and palate (CLP) who have undergone either orthodontic space closure or space opening for missing lateral incisors.

**Design:**

A cross-sectional retrospective study.

**Setting:**

University orthodontic clinic serving individuals with CLP.

**Patients:**

Twenty-eight individuals with nonsyndromic CLP who were missing lateral incisors divided into 2 groups: space closure (21 subjects) and space opening (7 subjects).

**Interventions:**

Orthodontic space closure or space opening for replacement of missing lateral incisors in individuals with nonsyndromic CLP.

**Main Outcome Measures:**

Buccal and palatal alveolar bone thickness were measured at 5 mm and 10 mm above the cementoenamel junction (CEJ) for cleft-adjacent central incisors and canines. Additionally, buccal, lingual, and proximal alveolar bony coverage ratio on cleft-side central incisors and canines were recorded.

**Results:**

No significant differences were observed in alveolar bone thickness and bony coverage between the space closure group and the space opening group, except for the buccal thickness 5 mm above the CEJ, which was thinner in the space closure group.

**Conclusion:**

The overall alveolar bone support in the grafted alveolus in both the space closure and space opening groups were comparable.

## Introduction

Cleft lip and palate (CLP) is among the most prevalent craniofacial anomalies, affecting approximately 1 to 7 newborns out of every 1000 births worldwide.^1^ CLP results from the incomplete fusion between the maxillary and medial nasal processes or between the palatal shelves or both during embryonic development, leading to a variety of structural deformities.^1,2^ More specifically, CLP can engender a multitude of dental anomalies, such as the absence of the maxillary lateral incisor, maxillary canine impaction, supernumerary teeth, ectopic eruption of the canine, and microdontia of the canine.^1–4^ However, one of the most common dental anomalies is agenesis of the maxillary lateral incisor.^1–5^

Maxillary lateral incisor agenesis in individuals with CLP often demands an interdisciplinary approach to restoring dental function and aesthetics.^5–9^ The main treatments used to address this issue are canine substitution and prosthetic replacements.^5–9^ The decision on which approach to employ involves evaluating several critical factors, such as complexity, long-term stability, cost, and aesthetics.^5–9^

Canine substitution, or space closure, entails the repositioning of canines to the grafted alveolar cleft with missing lateral incisors.^5–9^ The main advantage of this approach is that it is less invasive, preserving the adjacent teeth and soft tissue and promoting long-term stability of the area.^5–8^ It is also more cost-effective compared to prosthetic replacements.^8^ However, achieving good esthetics can be challenging.^5–7^ Typically, a series of restorative procedures on the cleft side canine and first premolar is needed to mimic the appearance of the lateral incisor and canine.^5–8^

In contrast to canine substitution, prosthetic replacements require space opening or maintenance. Potential prosthetic replacements for missing lateral incisors include resin-bonded fixed partial dentures, fixed partial dentures, single-tooth implants, and removable prostheses.^5,7,9,10^ Of all the replacement options, the most popular is single-tooth implants.^5,7,9,10^ A major advantage of single-tooth implants is that they do not impact adjacent teeth.^7,9,10^ However, producing a desirable esthetic outcome can be a strenuous process.^7,9,10^ Since the implant is placed in the esthetic zone, careful treatment planning and surgical procedures are crucial to achieving good esthetics.^7,9,10^ Furthermore, proper integration of the implant may require additional bone grafting in the alveolar ridge.^10–12^

Bone morphology near the cleft site must be considered when evaluating the success of both treatments. One of the goals of alveolar reconstruction is to facilitate eruption of permanent teeth adjacent to the cleft site with sound periodontal support.^12^ However, it remains unclear whether orthodontic tooth movement in a grafted alveolar cleft would provide sufficient alveolar support. Previous studies have compared the alveolar bone support of cleft-affected teeth with contralateral teeth on the noncleft side.^13,14^ However, there has been limited research on the differences in alveolar bone support among cleft adjacent teeth with different treatment options. This study evaluates the quality of alveolar bone adjacent to the cleft site by comparing space closure and space opening after bone grafting. Specifically, it compares the alveolar bone thickness and coverage of the cleft-side central incisor and canine between 2 conditions: when the canine was moved into the grafted alveolar cleft and when the space was further opened for future prosthodontic restoration. The findings will hopefully help clinicians make better decisions when treating maxillary lateral agenesis in CLP, improving patient outcomes.

## Subjects

Participants treated in the UCSF Orthodontics Department over the past decade were identified through the center's Epic database (IRB approval: 10-00564). Initial screening identified 180 individuals with CLP. The inclusion criteria for the study were: (1) nonsyndromic CLP, (2) presence of maxillary lateral incisor agenesis, (3) completion of alveolar bone grafting (ABG), (4) completion of orthodontic space opening or space closure treatment, (5) availability of cone beam computed tomography (CBCT) scans after Phase II. Individuals with syndromic CLP, supernumerary teeth at the cleft site, failed ABG, severe root resorption during orthodontic treatment, poor-quality CBCT scans, or who did not meet treatment criteria were excluded. After the screening, the final cohort consisted of 28 nonsyndromic CLP patients with missing one or both maxillary lateral incisors. Of these, 21 patients underwent space closure, while 7 underwent space opening. Demographic data for the study participants are presented in Table 1. The space closure group consisted of 12 male and 9 female patients, while the space opening group consisted of 4 male and 3 female patients. The ages at which CBCT was taken ranged from 15 to 29 years in the space closure group, and from 12 to 30 years in the space opening group. Cleft types included unilateral or bilateral CLP: 15 patients in the space closure group had UCLP and 6 had BCLP, while in the space opening group, 5 had UCLP and 2 had BCLP.

**Table 1.** Patient Demographic Characteristics.

	Space closure	Space opening
Patient number	21	7
Sex	12 male, 9 female	4 male, 3 female
Age at which CBCT was taken	15-29 years old	12-30 years old
Cleft type	15 unilateral (10 left, 5 right), 6 bilateral	5 unilateral (4 left, 1 right), 2 bilateral
Average duration between ABG and orthodontic treatment completion	7.20 years (0.55)	8.51 years (3.03)

CBCT, cone beam computed tomography; ABG, alveolar bone grafting.

Two surgeons at the same center performed all bone grafts using 100% autogenous iliac crest bone grafting material. The treatment protocol began with pregrafting Phase I orthodontics, which included maxillary arch expansion with a fan-shaped expander and upper incisor alignment using 2 × 4 mechanics. Following Phase I, patients underwent ABG before eruption of the canine adjacent to the cleft site. CBCT images were taken 4 to 6 months after graft consolidation to evaluate the graft outcome. After confirming the graft, patients were put into retention while awaiting readiness for Phase II orthodontics. After the completion of permanent dentition, Phase II treatment was initiated, involving comprehensive orthodontic treatment using 0.22-slot MBT brackets. During this phase, either space opening or space closure was performed. In the space closure group, spaces were closed with protracting the molars using a power chain on 0.018 stainless steel wire. After Phase II, brackets were removed, and a final round of CBCT imaging was performed. The CS 9300 Cone Beam Imaging System (Carestream Dental, Atlanta, CA) acquired all CBCT scans at an identical resolution.

The decision to pursue space closure versus space opening was based on several factors. Space closure was chosen when the gap between the central incisor and canine is relatively narrow (less than 6-7 mm), when the arch perimeter was not significantly compromised (such as in cases with minimal missing teeth), and when the smile line, midline, or gingival line remained intact. Additionally, financial considerations regarding the potential need for prosthodontic treatment influenced the decision.

## Methods for Evaluating the Bone Coverage and Thickness Near Cleft Site

**Figure 1.**
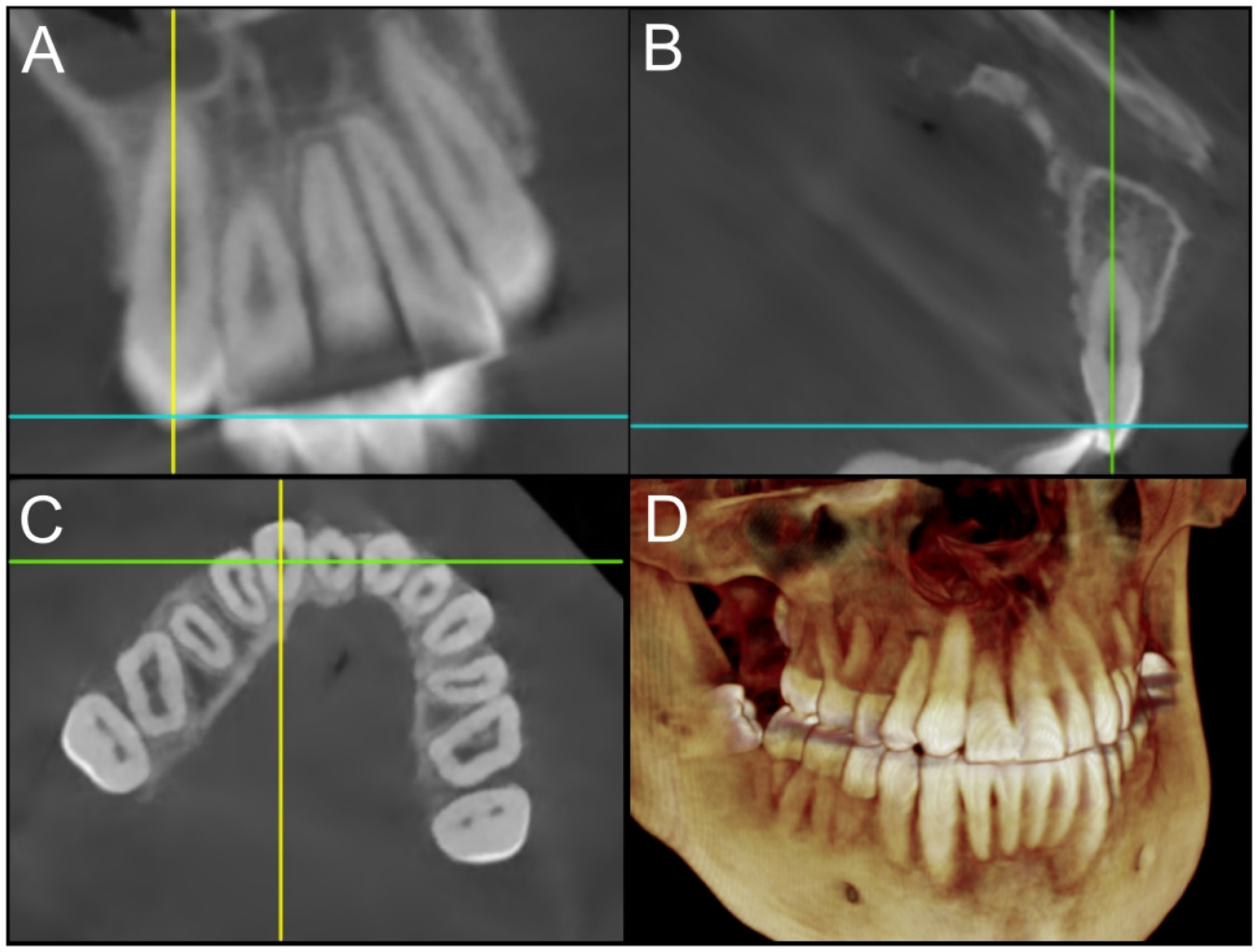
Orientation of CBCT scans along the long axis of the teeth: (A) sagittal plane parallel to the long axis; (B) coronal plane parallel to the long axis; (C) coronal plane parallel to a line passing through the centers of the canine and the central incisor; (D) 3D reconstruction view.

To assess the bone thickness and coverage adjacent to the cleft site for the space opening and space closure groups, we retrospectively analyzed the final CBCT scans taken after the completion of Phase II orthodontic treatment. The scans were loaded on Dolphin Imaging Plus (Dolphin Imaging and Management Solution, Chartworth, CA) and manually reoriented so that the long axis of the central incisor is parallel to the coronal and sagittal planes. On the axial plane, further adjustments were made so that the coronal plane was parallel to a line passing through the centers of the canine and the central incisor (Figure 1). This reorientation was applied separately to the central incisor and the canine.

CBCT, cone beam computed tomography.

**Figure 2.**
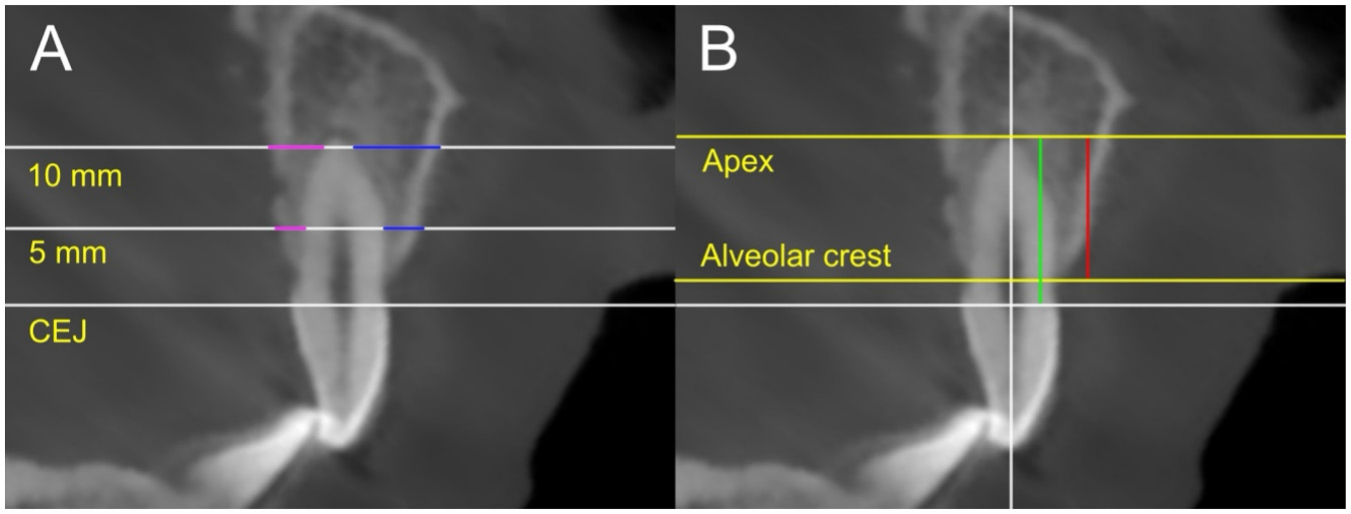
Measurement of alveolar bone thickness and alveolar root coverage: (A) the buccal (blue) and palatal (pink) thickness was measured 5 mm and 10 mm from the CEJ; (B) the percentage of the distance from the apex to the alveolar cleft (green) relative to the total root length (red) was calculated.

The alveolar bone thickness of the central incisor and canine adjacent to the cleft site was measured on both the buccal and palatal sides. Two horizontal reference lines, perpendicular to the coronal plane, were drawn at 5 mm and 10 mm from the cementoenamel junction (CEJ). These reference points were selected to represent bone thickness near the crown (5 mm) and deeper into the root (10 mm). Bone thickness was then measured along each line (Figure 2A). The buccal and palatal alveolar bone thicknesses were subsequently compared between groups.

CEJ, cementoenamel junction.

In addition, the alveolar bony coverage on the buccal, palatal, and cleft adjacent proximal surfaces of the central incisor and canine was measured. The buccal and palatal bony coverage was measured on the sagittal slice, and the proximal coverage was measured on the coronal slice (Figure 2B). On each surface, the CEJ and the point where alveolar bony coverage begins to disappear were identified. The distance between the apex and CEJ was measured as the root length, while the distance between the alveolar ridge and the apex was measured as the alveolar bony coverage. The percentage of bony coverage relative to root length was calculated and compared between groups.

## Statistical Analysis

The results were analyzed using Microsoft Excel (Microsoft Corporation, Redmond, WA, USA). Mean values were compared using an unpaired *t*-test between groups. A 95% confidence interval was applied, with the significance level set at *P* ≤ .05. All CBCT images were deidentified and coded to ensure blinding of investigators conducting the measurements. To assess interrater and intrarater reliability, a second investigator conducted assessments on 5 randomly selected patients, and the degree of agreement was evaluated using Pearson's coefficient, which was found to be 99.7%.

## Results

### Bone Thickness

Table 2 presents the mean buccal and palatal alveolar bone thickness of the central incisor and canine at 5 mm and 10 mm above the CEJ. The buccal alveolar bone thickness of the canine in the space closure group was statistically significantly smaller than in the space opening group (*P* = .0007*) at 5 mm above the CEJ. No other measurements showed statistically significant differences.

**Table 2.** Alveolar Bone Thickness (mm) and Root Bone Coverage (%).

	Buccal thickness (mm)				Palatal thickness (mm)				Buccal coverage (%)		Palatal coverage (%)		Proximal coverage (%)	
	Central incisor		Canine		Central incisor		Canine		Central incisor	Canine	Central incisor	Canine	Central incisor	Canine
Level	5 mm	10 mm	5 mm	10 mm	5 mm	10 mm	5 mm	10 mm						
Space closure	1.11 (0.86)	2.42 (1.35)	0.43 (0.60)	1.19 (0.95)	1.60 (1.31)	2.44 (1.92)	2.36 (1.43)	3.75 (2.12)	61.97 (26.22)	60.47 (26.03)	63.58 (32.69)	77.56 (18.67)	79.10 (11.44)	84.92 (4.96)
Space opening	1.43 (1.21)	2.70 (1.07)	1.9 (1.75)	1.90 (1.66)	1.32 (1.24)	2.31 (2.19)	3.31 (1.21)	4.71 (0.92)	68.59 (16.81)	69.59 (28.76)	63.58 (24.51)	85.45 (3.07)	74.46 (9.12)	81.93 (8.05)
*P*-value	N/S	N/S	.0007*	N/S	N/S	N/S	N/S	N/S	N/S	N/S	N/S	N/S	N/S	N/S

### Bone Coverage

Table 2 shows the mean alveolar bony coverage percentages on the buccal, palatal, and proximal surfaces of the central incisor and canine. No statistically significant differences were observed in any of the measurements.

## Discussion

Numerous studies indicate that CLP is associated with various dental anomalies, with the agenesis of the maxillary lateral incisor being the most commonly observed. A literature survey by Haque et al described congenitally missing teeth as a common dental anomaly related to CLP.^15^ A study by Paranaiba et al involving 296 Brazilian patients with nonsyndromic CLP found that 39.9% of the patients had at least one dental anomaly, with tooth agenesis identified as the most prevalent.^16^ Bartzela et al conducted a study specifically focused on tooth agenesis patterns in 115 unilateral CLP (UCLP) patients and reported 48.7% were missing at least one tooth, and the cleft side lateral incisor was the most frequently missing tooth.^4^ Furthermore, a retrospective analysis of patients with CLP by Al Jamal et al reinforced these findings, revealing that 66.7% of patients with CLP experienced tooth agenesis, with lateral incisors accounting for more than half of all missing teeth.^17^

Orthodontic space closure with canine substitution is a common approach for managing a missing lateral incisor. However, concerns remain regarding the adequacy of periodontal support due to various factors. Teeth adjacent to the cleft site have less alveolar bone support. Ercan et al observed that the buccal bone for cleft side central incisors was 0.18 mm thinner at the alveolar crest and 0.19 mm thinner at 2 mm apical of the crest compared to the contralateral incisor.^18^ Another study by Parveen et al found that the buccal bone thickness at 3 mm above the CEJ for teeth anterior to the cleft site was 0.7 mm thinner than the noncleft side.^19^ Furthermore, the results showed that bone 3 mm above the CEJ was 0.21 mm thinner at the distal surface of teeth anterior to the cleft and 0.35 mm thinner at the mesial surface of teeth posterior to the cleft in comparison to corresponding areas on the noncleft side.^19^ Additionally, Woods et al reported that the root surface of cleft-facing teeth had 5.7% less bony coverage compared to the contralateral teeth.^20^ Movahhedian et al observed that teeth anterior to the cleft had a root bone coverage ratio of 0.89, compared to 0.94 for the noncleft side, and also found that teeth anterior to the cleft site had a statistically higher number of fenestrations.^21^ Similarly, Buyuk et al reported a much higher prevalence of fenestrations and dehiscence in canines, lateral incisors, and central incisors on the cleft side compared to the noncleft side.^22^

In addition to the reduced periodontal support typically observed at the cleft site, further concerns arise regarding the periodontal support as the canine is orthodontically moved to the grafted alveolar cleft area. Several factors are considered when evaluating the success of an alveolar bone graft, including the presence of a cortical bony bridge, the vertical height or thickness of the bridge observed in radiographic images, and the clinical need for revision surgery.^23,24^ The reported success rate of ABG ranges from 86.2% to 94%.^25,26^

However, a successful bone graft does not necessarily lead to complete restoration of the alveolar cleft defect. Ko et al compared the total alveolar defect area with the actual grafted area 6 months to 1 year after ABG surgery, finding that bone fill ranged from 51.45% to 69.85%.^26^ Similarly, Liang et al reported a bone fill of 31.6% with rHBMP-2 grafting and 32.5% with autologous grafting.^27^ This is because the grafted bone undergoes extensive remodeling and transformation following alveolar bone graft surgery, with some of the grafted bone experiencing resorption.^28,29^ Nagashima et al reported a 48.8% resorption of grafted bone volume from 1 to 6 months postsurgery.^30^ In several other studies, the volume of the initial cleft defect was compared to the volume of the remaining grafted bone 1 year after surgery, with bony resorption ratios reported between 16% and 51%.^31–33^

This indicates that canine substitution entails moving the canine into the grafted alveolar cleft, which already presents with alveolar constriction. In the current study, we observed that the buccal bone thickness of the orthodontically repositioned canine, measured at 5 mm, was thinner than that of the cleft-side canine without space closure. This reduced thickness could be due to the canine moving into an anterior alveolus that already has a thinner bone structure, or it may be related to the canine being repositioned into a grafted area with inherently poorer alveolar conditions. These findings are consistent with those of Yatabe et al, who also reported thinner buccal alveolar bone in canines moved to a grafted site compared to the contralateral lateral incisors and canines.^13^ In their study, the buccal bone thickness of the cleft-side canine was recorded as 0.20 mm, with a palatal bone thickness of 1.39 mm, whereas the noncleft side tooth had a buccal bone thickness of 0.45 mm and a palatal bone thickness of 1.44 mm.^13^

Despite the statistically significant reduction in buccal alveolar thickness, the 0.43 mm of buccal bone thickness of the canine observed in the current study may not be clinically significant. Previous studies have reported that the buccal bone thickness of canines in noncleft patients is typically less than 1 mm.^34^ In addition, buccal alveolar bone coverage was observed in most canines in the current study, and no significant difference was found in the alveolar bony coverage ratio on the buccal surface of the canine root. It could mean that the buccal surface of the cleft canine, which was orthodontically moved into the grafted alveolus, was adequately protected by alveolar bone coverage, despite being thinner. Although there was no significant difference in the alveolar bone coverage between the space opening and space closure groups, this does not necessarily imply that the cleft-side incisors have adequate periodontal support comparable to the noncleft teeth. Woods et al observed that the cleft-side central incisors had 83.1% to 91.2% alveolar bone coverage and found that the cleft-facing surface of the root had 5.7% less alveolar bone coverage compared to the contralateral side, as mentioned earlier.^20^ Ercan also reported reduced bony support on the buccal surface which was consistent with the current study.^18^ This diminished support on the buccal surface may contribute to gingival recessions following compensatory orthodontic treatment, as reported by Zhu et al.^35^ Additionally, Garib et al suggested that the cleft-side canine typically exhibits more alveolar defects on the mesial surface than the distal surface.^36^ This indicates that clinicians should be aware that canines moved into the grafted cleft area may have reduced alveolar support.

Due to the retrospective nature of the study, it is challenging to draw definitive conclusions. Space closure and space opening were both influenced by clinical factors, which may have affected the outcomes. Additionally, the study is limited by its small sample size and the short-term nature of the data collected. Long-term bone resorption could potentially impact alveolar support over time. Further research is needed to fully understand how space opening and closure affect alveolar bone quality near the cleft site.

## Conclusion

The canine moved to the grafted alveolar cleft to replace the missing maxillary lateral incisor, demonstrating thinner buccal thickness at 5 mm above the CEJ. However, the overall alveolar bone support was comparable to that of the canine in its original location, as the difference was small and no significant difference in alveolar coverage was observed.
